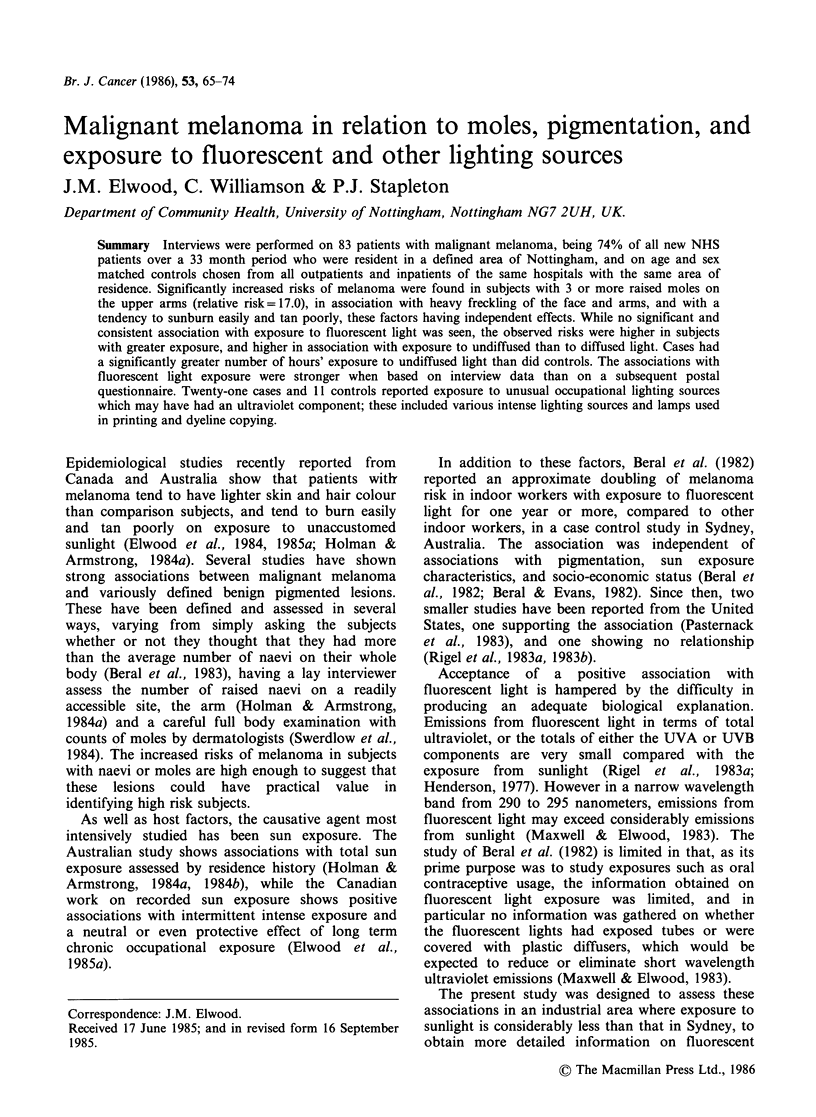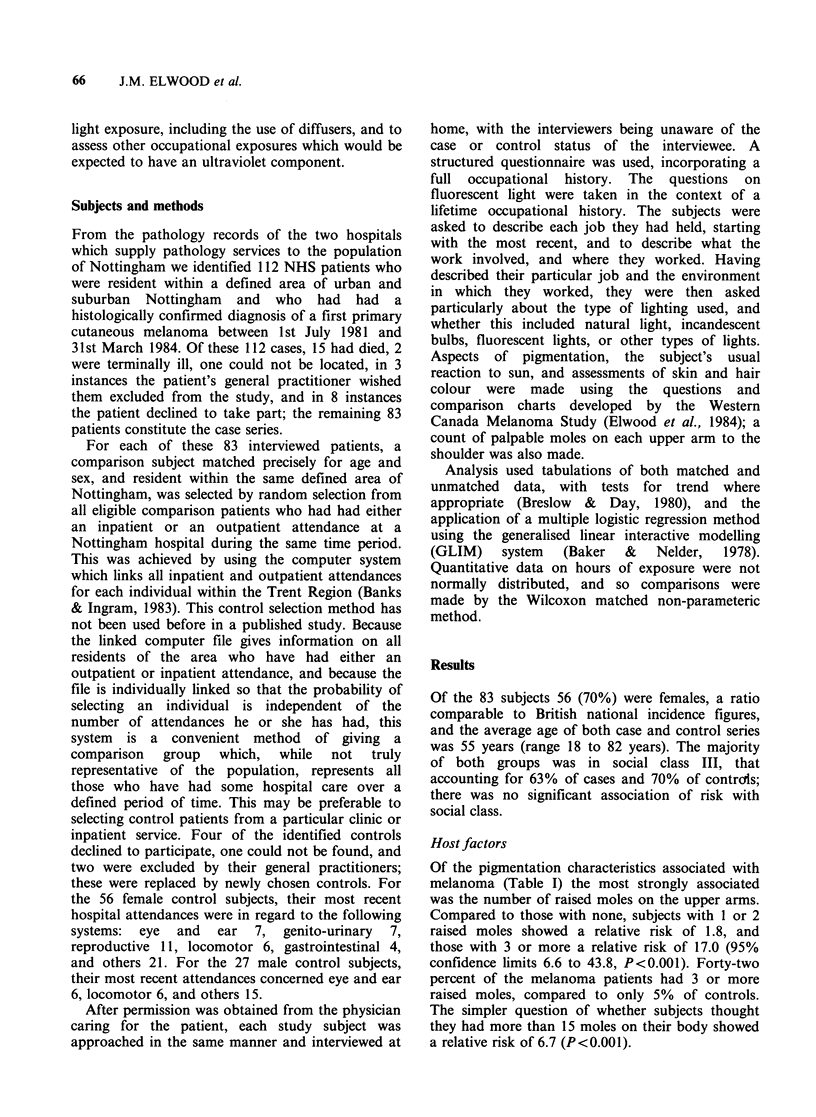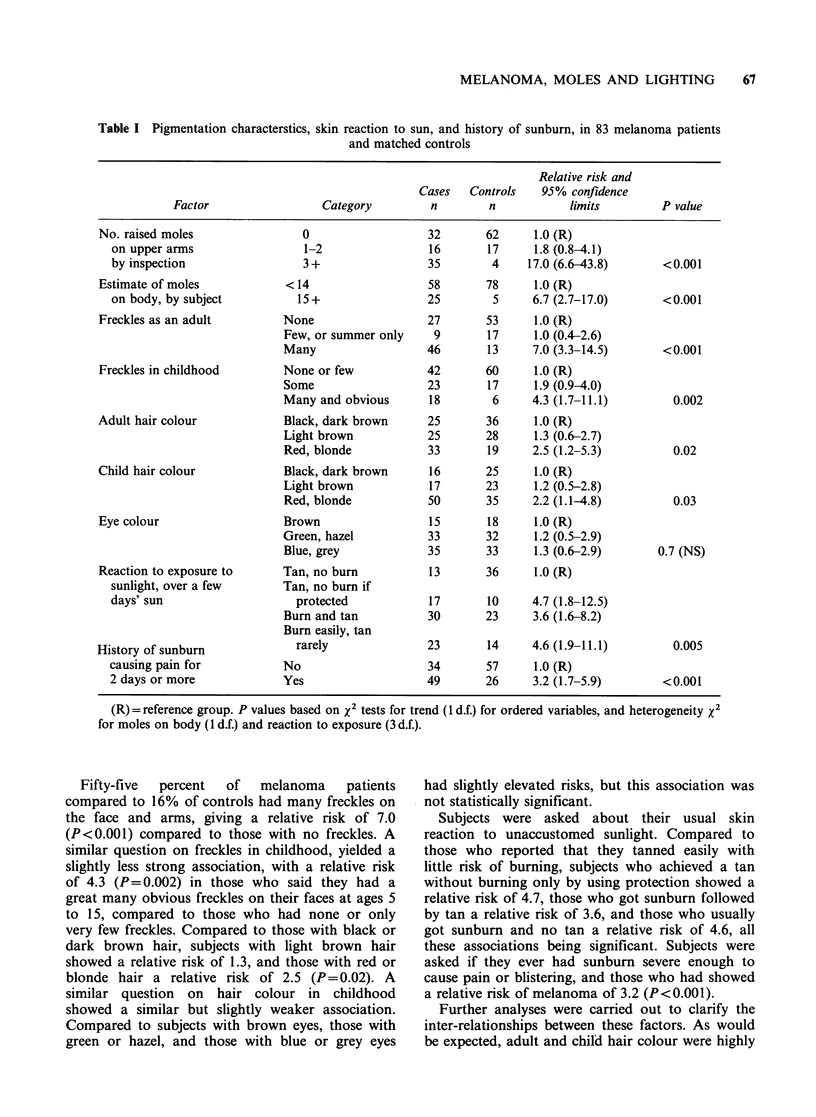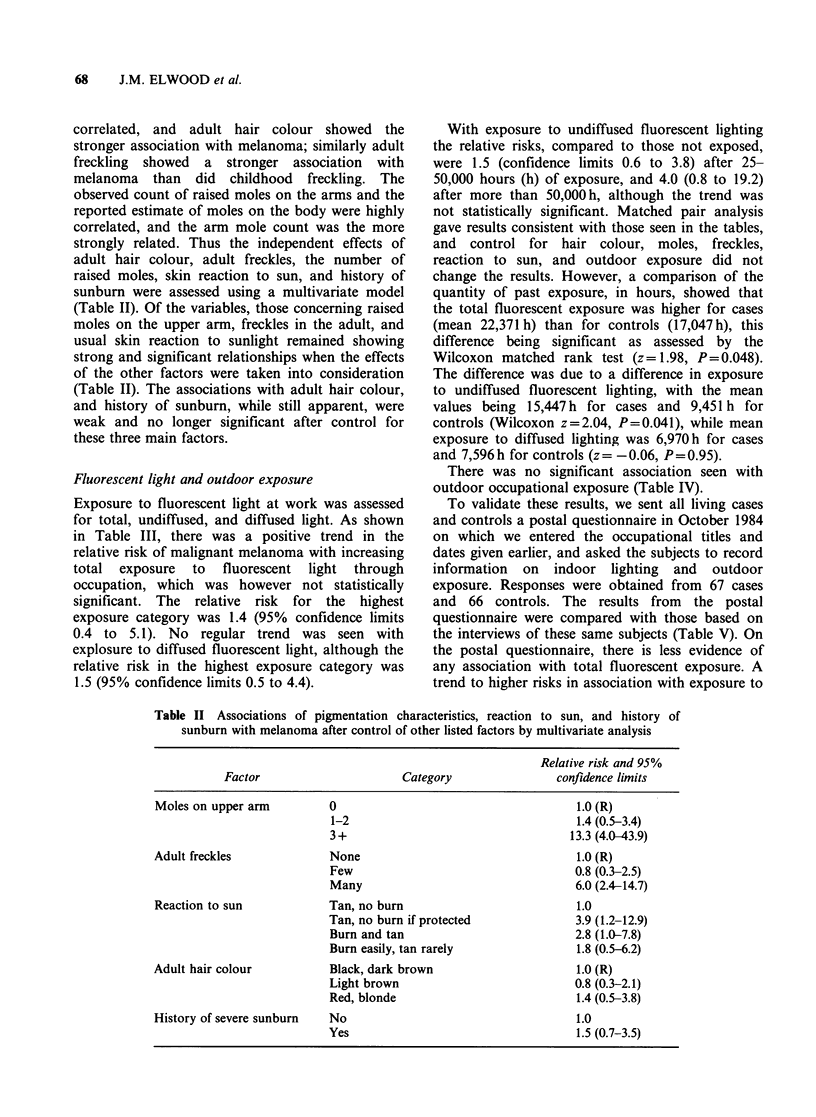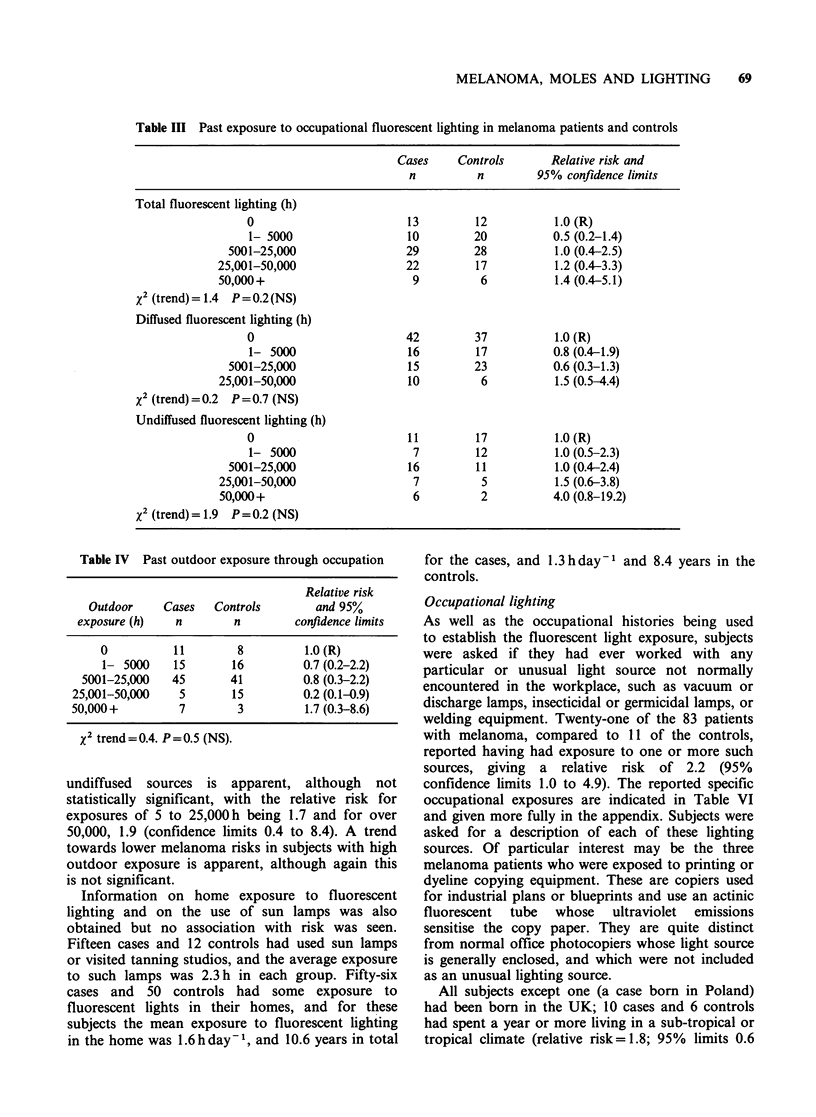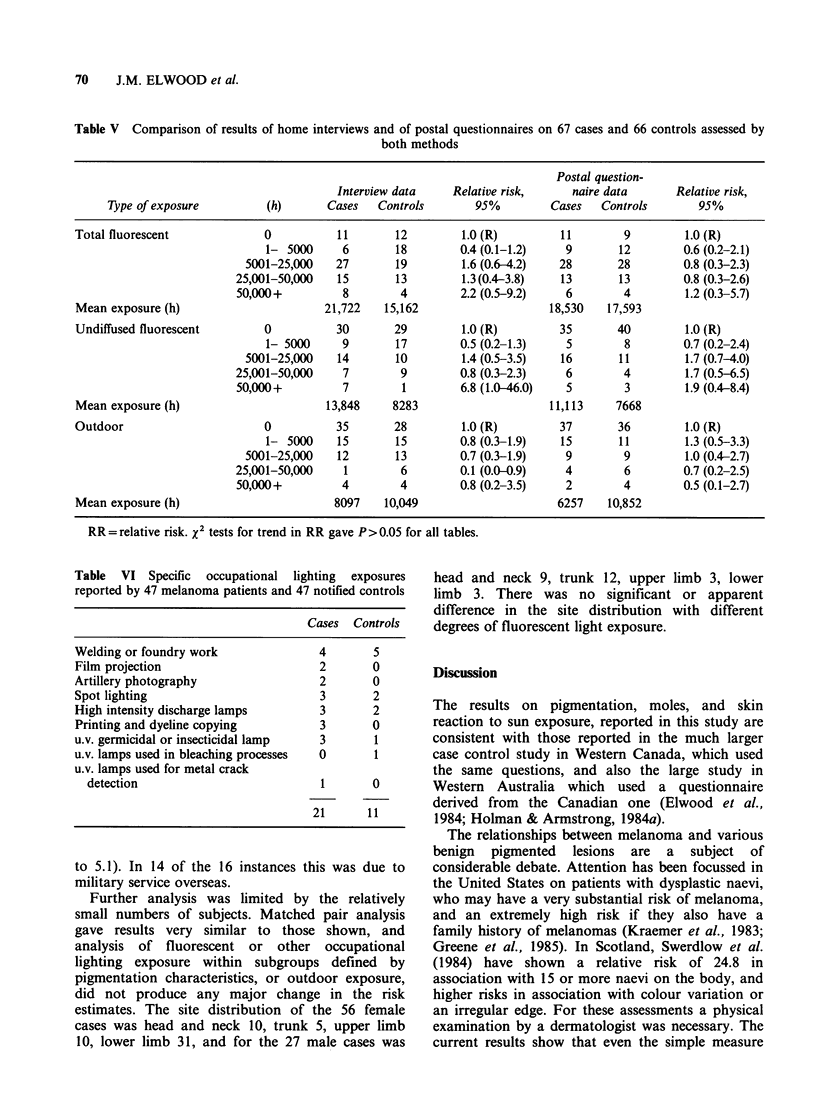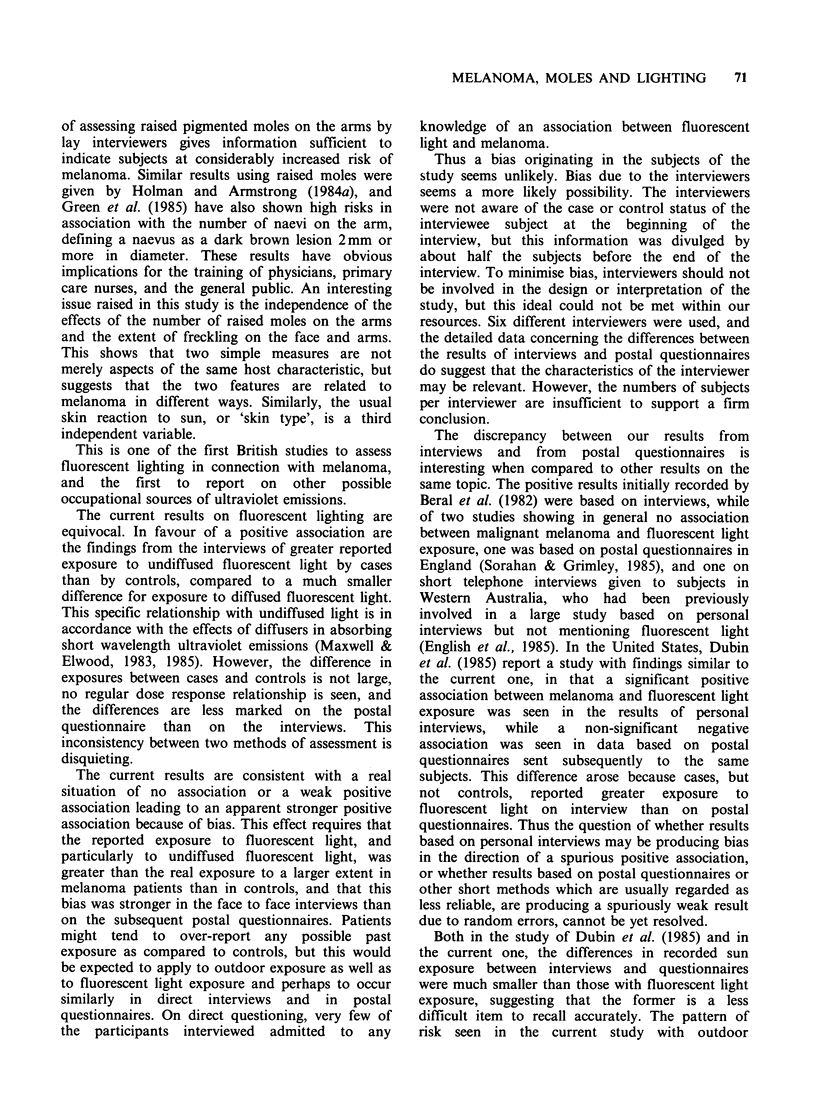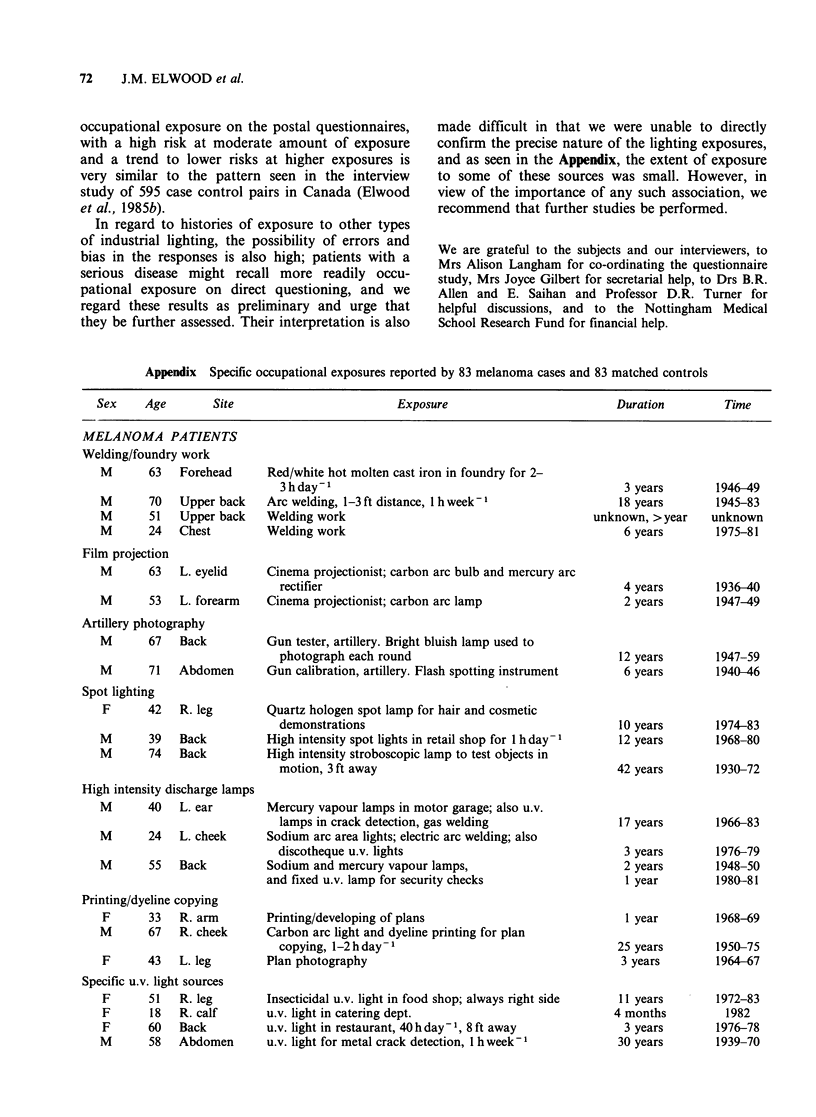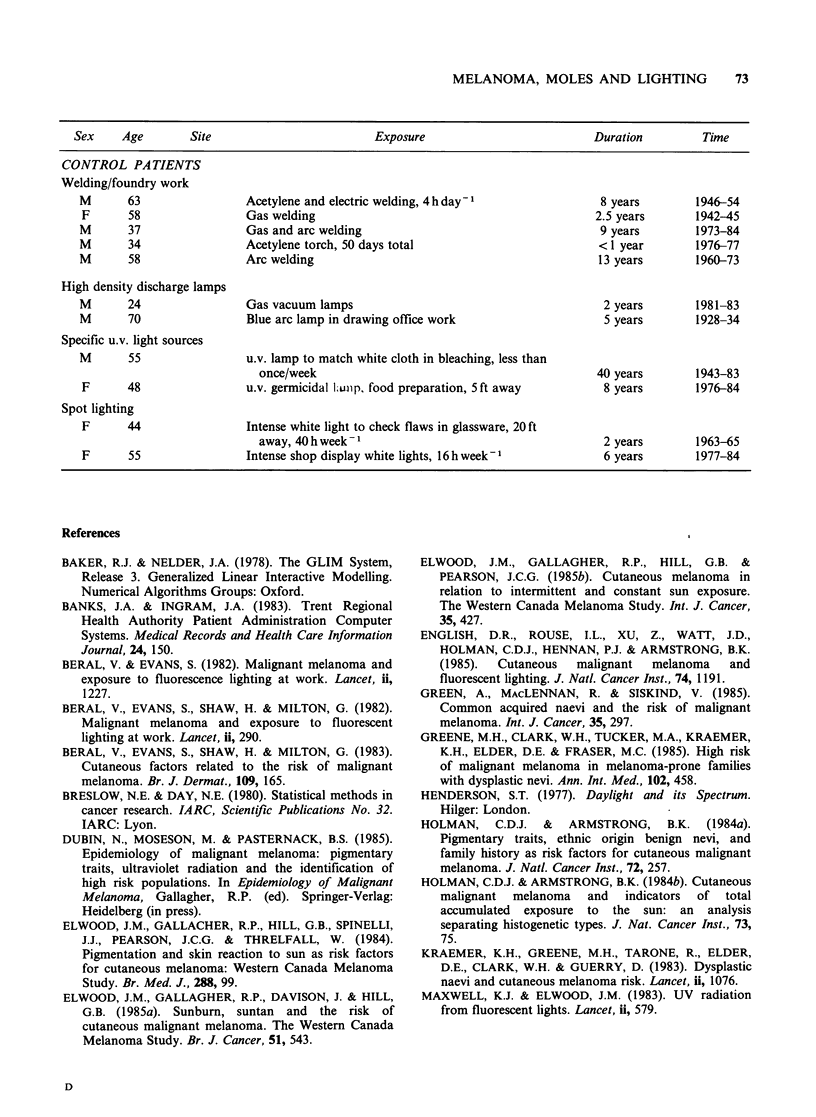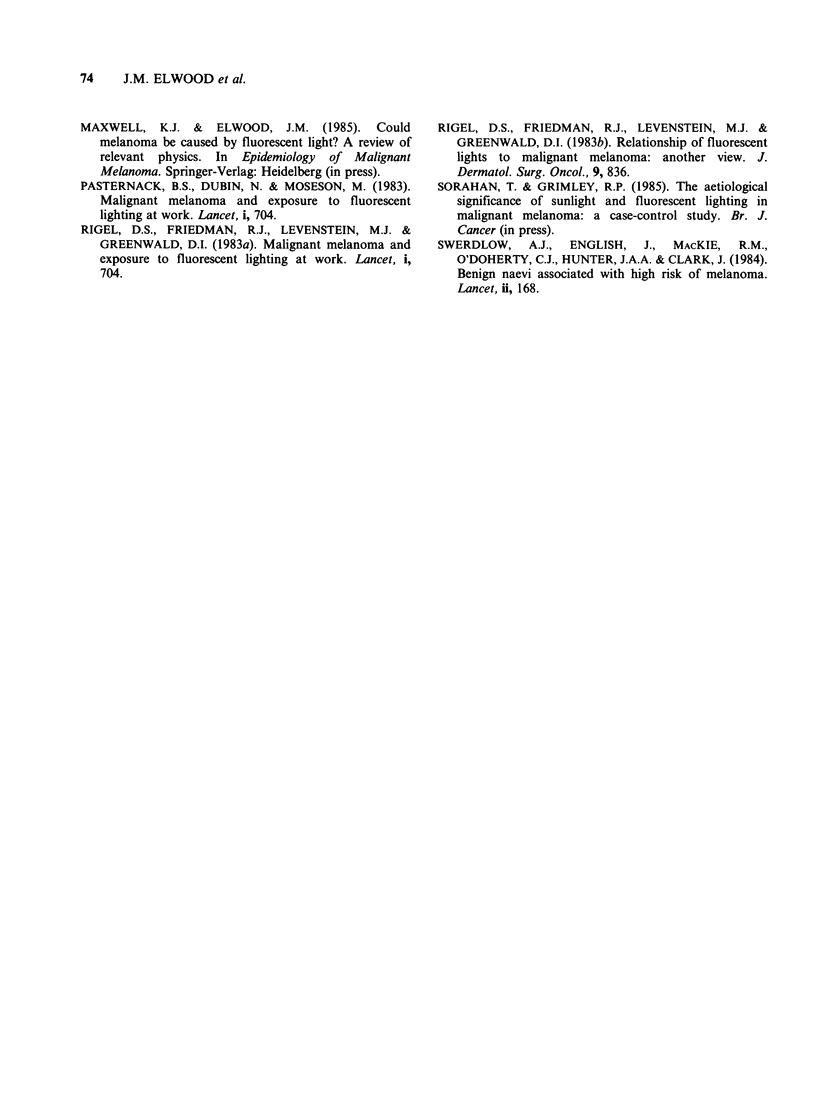# Malignant melanoma in relation to moles, pigmentation, and exposure to fluorescent and other lighting sources.

**DOI:** 10.1038/bjc.1986.10

**Published:** 1986-01

**Authors:** J. M. Elwood, C. Williamson, P. J. Stapleton

## Abstract

Interviews were performed on 83 patients with malignant melanoma, being 74% of all new NHS patients over a 33 month period who were resident in a defined area of Nottingham, and on age and sex matched controls chosen from all outpatients and inpatients of the same hospitals with the same area of residence. Significantly increased risks of melanoma were found in subjects with 3 or more raised moles on the upper arms (relative risk = 17.0), in association with heavy freckling of the face and arms, and with a tendency to sunburn easily and tan poorly, these factors having independent effects. While no significant and consistent association with exposure to fluorescent light was seen, the observed risks were higher in subjects with greater exposure, and higher in association with exposure to undiffused than to diffused light. Cases had a significantly greater number of hours' exposure to undiffused light than did controls. The associations with fluorescent light exposure were stronger when based on interview data than on a subsequent postal questionnaire. Twenty-one cases and 11 controls reported exposure to unusual occupational lighting sources which may have had an ultraviolet component; these included various intense lighting sources and lamps used in printing and dyeline copying.


*Br. J. Cancer* (1986), **53**, 65–74

### Malignant melanoma in relation to moles, pigmentation, and exposure to fluorescent and other lighting sources

J.M. Elwood, C. Williamson & P.J. Stapleton

*Department of Community Health, University of Nottingham, Nottingham NG7 2UH, UK.*

**Summary** Interviews were performed on 83 patients with malignant melanoma, being 74% of all new NHS patients over a 33 month period who were resident in a defined area of Nottingham, and on age and sex matched controls chosen from all outpatients and inpatients of the same hospitals with the same area of residence. Significantly increased risks of melanoma were found in subjects with 3 or more raised moles on the upper arms (relative risk = 17.0), in association with heavy freckling of the face and arms, and with a tendency to sunburn easily and tan poorly, these factors having independent effects. While no significant and consistent association with exposure to fluorescent light was seen, the observed risks were higher in subjects with greater exposure, and higher in association with exposure to undiffused than to diffused light. Cases had a significantly greater number of hours' exposure to undiffused light than did controls. The associations with fluorescent light exposure were stronger when based on interview data than on a subsequent postal questionnaire. Twenty-one cases and 11 controls reported exposure to unusual occupational lighting sources which may have had an ultraviolet component; these included various intense lighting sources and lamps used in printing and dyeline copying.

Epidemiological studies recently reported from Canada and Australia show that patients with melanoma tend to have lighter skin and hair colour than comparison subjects, and tend to burn easily and tan poorly on exposure to unaccustomed sunlight (Elwood *et al.*, 1984, 1985*a*; Holman & Armstrong, 1984*a*). Several studies have shown strong associations between malignant melanoma and variously defined benign pigmented lesions. These have been defined and assessed in several ways, varying from simply asking the subjects whether or not they thought that they had more than the average number of naevi on their whole body (Beral *et al.*, 1983), having a lay interviewer assess the number of raised naevi on a readily accessible site, the arm (Holman & Armstrong, 1984*a*) and a careful full body examination with counts of moles by dermatologists (Swerdlow *et al.*, 1984). The increased risks of melanoma in subjects with naevi or moles are high enough to suggest that these lesions could have practical value in identifying high risk subjects.

As well as host factors, the causative agent most intensively studied has been sun exposure. The Australian study shows associations with total sun exposure assessed by residence history (Holman & Armstrong, 1984*a*, 1984*b*), while the Canadian work on recorded sun exposure shows positive associations with intermittent intense exposure and a neutral or even protective effect of long term chronic occupational exposure (Elwood *et al.*, 1985*a*).

Correspondence: J.M. Elwood.
Received 17 June 1985; and in revised form 16 September 1985.

In addition to these factors, Beral *et al.* (1982) reported an approximate doubling of melanoma risk in indoor workers with exposure to fluorescent light for one year or more, compared to other indoor workers, in a case control study in Sydney, Australia. The association was independent of associations with pigmentation, sun exposure characteristics, and socio-economic status (Beral *et al.*, 1982; Beral & Evans, 1982). Since then, two smaller studies have been reported from the United States, one supporting the association (Pasternack *et al.*, 1983), and one showing no relationship (Rigel *et al.*, 1983*a*, 1983*b*).

Acceptance of a positive association with fluorescent light is hampered by the difficulty in producing an adequate biological explanation. Emissions from fluorescent light in terms of total ultraviolet, or the totals of either the UVA or UVB components are very small compared with the exposure from sunlight (Rigel *et al.*, 1983*a*; Henderson, 1977). However in a narrow wavelength band from 290 to 295 nanometers, emissions from fluorescent light may exceed considerably emissions from sunlight (Maxwell & Elwood, 1983). The study of Beral *et al.* (1982) is limited in that, as its prime purpose was to study exposures such as oral contraceptive usage, the information obtained on fluorescent light exposure was limited, and in particular no information was gathered on whether the fluorescent lights had exposed tubes or were covered with plastic diffusers, which would be expected to reduce or eliminate short wavelength ultraviolet emissions (Maxwell & Elwood, 1983).

The present study was designed to assess these associations in an industrial area where exposure to sunlight is considerably less than that in Sydney, to obtain more detailed information on fluorescent

© The Macmillan Press Ltd., 1986

light exposure, including the use of diffusers, and to assess other occupational exposures which would be expected to have an ultraviolet component.

### Subjects and methods

From the pathology records of the two hospitals which supply pathology services to the population of Nottingham we identified 112 NHS patients who were resident within a defined area of urban and suburban Nottingham and who had had a histologically confirmed diagnosis of a first primary cutaneous melanoma between 1st July 1981 and 31st March 1984. Of these 112 cases, 15 had died, 2 were terminally ill, one could not be located, in 3 instances the patient's general practitioner wished them excluded from the study, and in 8 instances the patient declined to take part; the remaining 83 patients constitute the case series.

For each of these 83 interviewed patients, a comparison subject matched precisely for age and sex, and resident within the same defined area of Nottingham, was selected by random selection from all eligible comparison patients who had had either an inpatient or an outpatient attendance at a Nottingham hospital during the same time period. This was achieved by using the computer system which links all inpatient and outpatient attendances for each individual within the Trent Region (Banks & Ingram, 1983). This control selection method has not been used before in a published study. Because the linked computer file gives information on all residents of the area who have had either an outpatient or inpatient attendance, and because the file is individually linked so that the probability of selecting an individual is independent of the number of attendances he or she has had, this system is a convenient method of giving a comparison group which, while not truly representative of the population, represents all those who have had some hospital care over a defined period of time. This may be preferable to selecting control patients from a particular clinic or inpatient service. Four of the identified controls declined to participate, one could not be found, and two were excluded by their general practitioners; these were replaced by newly chosen controls. For the 56 female control subjects, their most recent hospital attendances were in regard to the following systems: eye and ear 7, genito-urinary 7, reproductive 11, locomotor 6, gastrointestinal 4, and others 21. For the 27 male control subjects, their most recent attendances concerned eye and ear 6, locomotor 6, and others 15.

After permission was obtained from the physician caring for the patient, each study subject was approached in the same manner and interviewed at home, with the interviewers being unaware of the case or control status of the interviewee. A structured questionnaire was used, incorporating a full occupational history. The questions on fluorescent light were taken in the context of a lifetime occupational history. The subjects were asked to describe each job they had held, starting with the most recent, and to describe what the work involved, and where they worked. Having described their particular job and the environment in which they worked, they were then asked particularly about the type of lighting used, and whether this included natural light, incandescent bulbs, fluorescent lights, or other types of lights. Aspects of pigmentation, the subject's usual reaction to sun, and assessments of skin and hair colour were made using the questions and comparison charts developed by the Western Canada Melanoma Study (Elwood *et al.*, 1984); a count of palpable moles on each upper arm to the shoulder was also made.

Analysis used tabulations of both matched and unmatched data, with tests for trend where appropriate (Breslow & Day, 1980), and the application of a multiple logistic regression method using the generalised linear interactive modelling (GLIM) system (Baker & Nelder, 1978). Quantitative data on hours of exposure were not normally distributed, and so comparisons were made by the Wilcoxon matched non-parameteric method.

### Results

Of the 83 subjects 56 (70%) were females, a ratio comparable to British national incidence figures, and the average age of both case and control series was 55 years (range 18 to 82 years). The majority of both groups was in social class III, that accounting for 63% of cases and 70% of controls; there was no significant association of risk with social class.

### *Host factors*

Of the pigmentation characteristics associated with melanoma (Table I) the most strongly associated was the number of raised moles on the upper arms. Compared to those with none, subjects with 1 or 2 raised moles showed a relative risk of 1.8, and those with 3 or more a relative risk of 17.0 (95% confidence limits 6.6 to 43.8, $P<0.001$). Forty-two percent of the melanoma patients had 3 or more raised moles, compared to only 5% of controls. The simpler question of whether subjects thought they had more than 15 moles on their body showed a relative risk of 6.7 ($P<0.001$).

**Table I** Pigmentation characterstics, skin reaction to sun, and history of sunburn, in 83 melanoma patients and matched controls

| *Factor* | *Category* | *Cases n* | *Controls n* | *Relative risk and 95% confidence limits* | *P value* |
|---|---|---|---|---|---|
| No. raised moles on upper arms by inspection | 0 | 32 | 62 | 1.0 (R) | |
| | 1–2 | 16 | 17 | 1.8 (0.8–4.1) | |
| | 3+ | 35 | 4 | 17.0 (6.6–43.8) | <0.001 |
| Estimate of moles on body, by subject | <14 | 58 | 78 | 1.0 (R) | |
| | 15+ | 25 | 5 | 6.7 (2.7–17.0) | <0.001 |
| Freckles as an adult | None | 27 | 53 | 1.0 (R) | |
| | Few, or summer only | 9 | 17 | 1.0 (0.4–2.6) | |
| | Many | 46 | 13 | 7.0 (3.3–14.5) | <0.001 |
| Freckles in childhood | None or few | 42 | 60 | 1.0 (R) | |
| | Some | 23 | 17 | 1.9 (0.9–4.0) | |
| | Many and obvious | 18 | 6 | 4.3 (1.7–11.1) | 0.002 |
| Adult hair colour | Black, dark brown | 25 | 36 | 1.0 (R) | |
| | Light brown | 25 | 28 | 1.3 (0.6–2.7) | |
| | Red, blonde | 33 | 19 | 2.5 (1.2–5.3) | 0.02 |
| Child hair colour | Black, dark brown | 16 | 25 | 1.0 (R) | |
| | Light brown | 17 | 23 | 1.2 (0.5–2.8) | |
| | Red, blonde | 50 | 35 | 2.2 (1.1–4.8) | 0.03 |
| Eye colour | Brown | 15 | 18 | 1.0 (R) | |
| | Green, hazel | 33 | 32 | 1.2 (0.5–2.9) | |
| | Blue, grey | 35 | 33 | 1.3 (0.6–2.9) | 0.7 (NS) |
| Reaction to exposure to sunlight, over a few days' sun | Tan, no burn | 13 | 36 | 1.0 (R) | |
| | Tan, no burn if protected | 17 | 10 | 4.7 (1.8–12.5) | |
| | Burn and tan | 30 | 23 | 3.6 (1.6–8.2) | |
| | Burn easily, tan rarely | 23 | 14 | 4.6 (1.9–11.1) | 0.005 |
| History of sunburn causing pain for 2 days or more | No | 34 | 57 | 1.0 (R) | |
| | Yes | 49 | 26 | 3.2 (1.7–5.9) | <0.001 |

(R) = reference group. *P* values based on $\chi^2$ tests for trend (1 d.f.) for ordered variables, and heterogeneity $\chi^2$ for moles on body (1 d.f.) and reaction to exposure (3 d.f.).

Fifty-five percent of melanoma patients compared to 16% of controls had many freckles on the face and arms, giving a relative risk of 7.0 ($P<0.001$) compared to those with no freckles. A similar question on freckles in childhood, yielded a slightly less strong association, with a relative risk of 4.3 ($P=0.002$) in those who said they had a great many obvious freckles on their faces at ages 5 to 15, compared to those who had none or only very few freckles. Compared to those with black or dark brown hair, subjects with light brown hair showed a relative risk of 1.3, and those with red or blonde hair a relative risk of 2.5 ($P=0.02$). A similar question on hair colour in childhood showed a similar but slightly weaker association. Compared to subjects with brown eyes, those with green or hazel, and those with blue or grey eyes had slightly elevated risks, but this association was not statistically significant.

Subjects were asked about their usual skin reaction to unaccustomed sunlight. Compared to those who reported that they tanned easily with little risk of burning, subjects who achieved a tan without burning only by using protection showed a relative risk of 4.7, those who got sunburn followed by tan a relative risk of 3.6, and those who usually got sunburn and no tan a relative risk of 4.6, all these associations being significant. Subjects were asked if they ever had sunburn severe enough to cause pain or blistering, and those who had showed a relative risk of melanoma of 3.2 ($P<0.001$).

Further analyses were carried out to clarify the inter-relationships between these factors. As would be expected, adult and child hair colour were highly correlated, and adult hair colour showed the stronger association with melanoma; similarly adult freckling showed a stronger association with melanoma than did childhood freckling. The observed count of raised moles on the arms and the reported estimate of moles on the body were highly correlated, and the arm mole count was the more strongly related. Thus the independent effects of adult hair colour, adult freckles, the number of raised moles, skin reaction to sun, and history of sunburn were assessed using a multivariate model (Table II). Of the variables, those concerning raised moles on the upper arm, freckles in the adult, and usual skin reaction to sunlight remained showing strong and significant relationships when the effects of the other factors were taken into consideration (Table II). The associations with adult hair colour, and history of sunburn, while still apparent, were weak and no longer significant after control for these three main factors.

*Fluorescent light and outdoor exposure*

Exposure to fluorescent light at work was assessed for total, undiffused, and diffused light. As shown in Table III, there was a positive trend in the relative risk of malignant melanoma with increasing total exposure to fluorescent light through occupation, which was however not statistically significant. The relative risk for the highest exposure category was 1.4 (95% confidence limits 0.4 to 5.1). No regular trend was seen with explosure to diffused fluorescent light, although the relative risk in the highest exposure category was 1.5 (95% confidence limits 0.5 to 4.4).

With exposure to undiffused fluorescent lighting the relative risks, compared to those not exposed, were 1.5 (confidence limits 0.6 to 3.8) after 25–50,000 hours (h) of exposure, and 4.0 (0.8 to 19.2) after more than 50,000 h, although the trend was not statistically significant. Matched pair analysis gave results consistent with those seen in the tables, and control for hair colour, moles, freckles, reaction to sun, and outdoor exposure did not change the results. However, a comparison of the quantity of past exposure, in hours, showed that the total fluorescent exposure was higher for cases (mean 22,371 h) than for controls (17,047 h), this difference being significant as assessed by the Wilcoxon matched rank test ($z = 1.98$, $P = 0.048$). The difference was due to a difference in exposure to undiffused fluorescent lighting, with the mean values being 15,447 h for cases and 9,451 h for controls (Wilcoxon $z = 2.04$, $P = 0.041$), while mean exposure to diffused lighting was 6,970 h for cases and 7,596 h for controls ($z = -0.06$, $P = 0.95$).

There was no significant association seen with outdoor occupational exposure (Table IV).

To validate these results, we sent all living cases and controls a postal questionnaire in October 1984 on which we entered the occupational titles and dates given earlier, and asked the subjects to record information on indoor lighting and outdoor exposure. Responses were obtained from 67 cases and 66 controls. The results from the postal questionnaire were compared with those based on the interviews of these same subjects (Table V). On the postal questionnaire, there is less evidence of any association with total fluorescent exposure. A trend to higher risks in association with exposure to

**Table II** Associations of pigmentation characteristics, reaction to sun, and history of sunburn with melanoma after control of other listed factors by multivariate analysis

| *Factor* | *Category* | *Relative risk and 95% confidence limits* |
|---|---|---|
| Moles on upper arm | 0 | 1.0 (R) |
| | 1–2 | 1.4 (0.5–3.4) |
| | 3+ | 13.3 (4.0–43.9) |
| Adult freckles | None | 1.0 (R) |
| | Few | 0.8 (0.3–2.5) |
| | Many | 6.0 (2.4–14.7) |
| Reaction to sun | Tan, no burn | 1.0 |
| | Tan, no burn if protected | 3.9 (1.2–12.9) |
| | Burn and tan | 2.8 (1.0–7.8) |
| | Burn easily, tan rarely | 1.8 (0.5–6.2) |
| Adult hair colour | Black, dark brown | 1.0 (R) |
| | Light brown | 0.8 (0.3–2.1) |
| | Red, blonde | 1.4 (0.5–3.8) |
| History of severe sunburn | No | 1.0 |
| | Yes | 1.5 (0.7–3.5) |

**Table III** Past exposure to occupational fluorescent lighting in melanoma patients and controls

| | *Cases* *n* | *Controls* *n* | *Relative risk and 95% confidence limits* |
|---|---|---|---|
| Total fluorescent lighting (h) | | | |
| 0 | 13 | 12 | 1.0 (R) |
| 1- 5000 | 10 | 20 | 0.5 (0.2–1.4) |
| 5001–25,000 | 29 | 28 | 1.0 (0.4–2.5) |
| 25,001–50,000 | 22 | 17 | 1.2 (0.4–3.3) |
| 50,000+ | 9 | 6 | 1.4 (0.4–5.1) |
| $\chi^2$ (trend) = 1.4 $P$ = 0.2 (NS) | | | |
| Diffused fluorescent lighting (h) | | | |
| 0 | 42 | 37 | 1.0 (R) |
| 1- 5000 | 16 | 17 | 0.8 (0.4–1.9) |
| 5001–25,000 | 15 | 23 | 0.6 (0.3–1.3) |
| 25,001–50,000 | 10 | 6 | 1.5 (0.5–4.4) |
| $\chi^2$ (trend) = 0.2 $P$ = 0.7 (NS) | | | |
| Undiffused fluorescent lighting (h) | | | |
| 0 | 11 | 17 | 1.0 (R) |
| 1- 5000 | 7 | 12 | 1.0 (0.5–2.3) |
| 5001–25,000 | 16 | 11 | 1.0 (0.4–2.4) |
| 25,001–50,000 | 7 | 5 | 1.5 (0.6–3.8) |
| 50,000+ | 6 | 2 | 4.0 (0.8–19.2) |
| $\chi^2$ (trend) = 1.9 $P$ = 0.2 (NS) | | | |

**Table IV** Past outdoor exposure through occupation

| *Outdoor exposure (h)* | *Cases* *n* | *Controls* *n* | *Relative risk and 95% confidence limits* |
|---|---|---|---|
| 0 | 11 | 8 | 1.0 (R) |
| 1- 5000 | 15 | 16 | 0.7 (0.2–2.2) |
| 5001–25,000 | 45 | 41 | 0.8 (0.3–2.2) |
| 25,001–50,000 | 5 | 15 | 0.2 (0.1–0.9) |
| 50,000+ | 7 | 3 | 1.7 (0.3–8.6) |

$\chi^2$ trend = 0.4. $P$ = 0.5 (NS).

undiffused sources is apparent, although not statistically significant, with the relative risk for exposures of 5 to 25,000 h being 1.7 and for over 50,000, 1.9 (confidence limits 0.4 to 8.4). A trend towards lower melanoma risks in subjects with high outdoor exposure is apparent, although again this is not significant.

Information on home exposure to fluorescent lighting and on the use of sun lamps was also obtained but no association with risk was seen. Fifteen cases and 12 controls had used sun lamps or visited tanning studios, and the average exposure to such lamps was 2.3 h in each group. Fifty-six cases and 50 controls had some exposure to fluorescent lights in their homes, and for these subjects the mean exposure to fluorescent lighting in the home was 1.6 h $day^{-1}$, and 10.6 years in total for the cases, and 1.3 h $day^{-1}$ and 8.4 years in the controls.

*Occupational lighting*

As well as the occupational histories being used to establish the fluorescent light exposure, subjects were asked if they had ever worked with any particular or unusual light source not normally encountered in the workplace, such as vacuum or discharge lamps, insecticidal or germicidal lamps, or welding equipment. Twenty-one of the 83 patients with melanoma, compared to 11 of the controls, reported having had exposure to one or more such sources, giving a relative risk of 2.2 (95% confidence limits 1.0 to 4.9). The reported specific occupational exposures are indicated in Table VI and given more fully in the appendix. Subjects were asked for a description of each of these lighting sources. Of particular interest may be the three melanoma patients who were exposed to printing or dyeline copying equipment. These are copiers used for industrial plans or blueprints and use an actinic fluorescent tube whose ultraviolet emissions sensitise the copy paper. They are quite distinct from normal office photocopiers whose light source is generally enclosed, and which were not included as an unusual lighting source.

All subjects except one (a case born in Poland) had been born in the UK; 10 cases and 6 controls had spent a year or more living in a sub-tropical or tropical climate (relative risk = 1.8; 95% limits 0.6

**Table V** Comparison of results of home interviews and of postal questionnaires on 67 cases and 66 controls assessed by both methods

| *Type of exposure* | (*h*) | *Interview data* Cases | *Interview data* Controls | *Relative risk, 95%* | *Postal questionnaire data* Cases | *Postal questionnaire data* Controls | *Relative risk, 95%* |
|---|---|---|---|---|---|---|---|
| Total fluorescent | 0 | 11 | 12 | 1.0 (R) | 11 | 9 | 1.0 (R) |
| | 1- 5000 | 6 | 18 | 0.4 (0.1–1.2) | 9 | 12 | 0.6 (0.2–2.1) |
| | 5001–25,000 | 27 | 19 | 1.6 (0.6–4.2) | 28 | 28 | 0.8 (0.3–2.3) |
| | 25,001–50,000 | 15 | 13 | 1.3 (0.4–3.8) | 13 | 13 | 0.8 (0.3–2.6) |
| | 50,000+ | 8 | 4 | 2.2 (0.5–9.2) | 6 | 4 | 1.2 (0.3–5.7) |
| Mean exposure (h) | | 21,722 | 15,162 | | 18,530 | 17,593 | |
| Undiffused fluorescent | 0 | 30 | 29 | 1.0 (R) | 35 | 40 | 1.0 (R) |
| | 1- 5000 | 9 | 17 | 0.5 (0.2–1.3) | 5 | 8 | 0.7 (0.2–2.4) |
| | 5001–25,000 | 14 | 10 | 1.4 (0.5–3.5) | 16 | 11 | 1.7 (0.7–4.0) |
| | 25,001–50,000 | 7 | 9 | 0.8 (0.3–2.3) | 6 | 4 | 1.7 (0.5–6.5) |
| | 50,000+ | 7 | 1 | 6.8 (1.0–46.0) | 5 | 3 | 1.9 (0.4–8.4) |
| Mean exposure (h) | | 13,848 | 8283 | | 11,113 | 7668 | |
| Outdoor | 0 | 35 | 28 | 1.0 (R) | 37 | 36 | 1.0 (R) |
| | 1- 5000 | 15 | 15 | 0.8 (0.3–1.9) | 15 | 11 | 1.3 (0.5–3.3) |
| | 5001–25,000 | 12 | 13 | 0.7 (0.3–1.9) | 9 | 9 | 1.0 (0.4–2.7) |
| | 25,001–50,000 | 1 | 6 | 0.1 (0.0–0.9) | 4 | 6 | 0.7 (0.2–2.5) |
| | 50,000+ | 4 | 4 | 0.8 (0.2–3.5) | 2 | 4 | 0.5 (0.1–2.7) |
| Mean exposure (h) | | 8097 | 10,049 | | 6257 | 10,852 | |

RR = relative risk. $\chi^2$ tests for trend in RR gave $P > 0.05$ for all tables.

**Table VI** Specific occupational lighting exposures reported by 47 melanoma patients and 47 notified controls

| | *Cases* | *Controls* |
|---|---|---|
| Welding or foundry work | 4 | 5 |
| Film projection | 2 | 0 |
| Artillery photography | 2 | 0 |
| Spot lighting | 3 | 2 |
| High intensity discharge lamps | 3 | 2 |
| Printing and dyeline copying | 3 | 0 |
| u.v. germicidal or insecticidal lamp | 3 | 1 |
| u.v. lamps used in bleaching processes | 0 | 1 |
| u.v. lamps used for metal crack detection | 1 | 0 |
| | 21 | 11 |

to 5.1). In 14 of the 16 instances this was due to military service overseas.

Further analysis was limited by the relatively small numbers of subjects. Matched pair analysis gave results very similar to those shown, and analysis of fluorescent or other occupational lighting exposure within subgroups defined by pigmentation characteristics, or outdoor exposure, did not produce any major change in the risk estimates. The site distribution of the 56 female cases was head and neck 10, trunk 5, upper limb 10, lower limb 31, and for the 27 male cases was head and neck 9, trunk 12, upper limb 3, lower limb 3. There was no significant or apparent difference in the site distribution with different degrees of fluorescent light exposure.

### Discussion

The results on pigmentation, moles, and skin reaction to sun exposure, reported in this study are consistent with those reported in the much larger case control study in Western Canada, which used the same questions, and also the large study in Western Australia which used a questionnaire derived from the Canadian one (Elwood *et al.*, 1984; Holman & Armstrong, 1984*a*).

The relationships between melanoma and various benign pigmented lesions are a subject of considerable debate. Attention has been focussed in the United States on patients with dysplastic naevi, who may have a very substantial risk of melanoma, and an extremely high risk if they also have a family history of melanomas (Kraemer *et al.*, 1983; Greene *et al.*, 1985). In Scotland, Swerdlow *et al.* (1984) have shown a relative risk of 24.8 in association with 15 or more naevi on the body, and higher risks in association with colour variation or an irregular edge. For these assessments a physical examination by a dermatologist was necessary. The current results show that even the simple measure of assessing raised pigmented moles on the arms by lay interviewers gives information sufficient to indicate subjects at considerably increased risk of melanoma. Similar results using raised moles were given by Holman and Armstrong (1984*a*), and Green *et al.* (1985) have also shown high risks in association with the number of naevi on the arm, defining a naevus as a dark brown lesion 2 mm or more in diameter. These results have obvious implications for the training of physicians, primary care nurses, and the general public. An interesting issue raised in this study is the independence of the effects of the number of raised moles on the arms and the extent of freckling on the face and arms. This shows that two simple measures are not merely aspects of the same host characteristic, but suggests that the two features are related to melanoma in different ways. Similarly, the usual skin reaction to sun, or 'skin type', is a third independent variable.

This is one of the first British studies to assess fluorescent lighting in connection with melanoma, and the first to report on other possible occupational sources of ultraviolet emissions.

The current results on fluorescent lighting are equivocal. In favour of a positive association are the findings from the interviews of greater reported exposure to undiffused fluorescent light by cases than by controls, compared to a much smaller difference for exposure to diffused fluorescent light. This specific relationship with undiffused light is in accordance with the effects of diffusers in absorbing short wavelength ultraviolet emissions (Maxwell & Elwood, 1983, 1985). However, the difference in exposures between cases and controls is not large, no regular dose response relationship is seen, and the differences are less marked on the postal questionnaire than on the interviews. This inconsistency between two methods of assessment is disquieting.

The current results are consistent with a real situation of no association or a weak positive association leading to an apparent stronger positive association because of bias. This effect requires that the reported exposure to fluorescent light, and particularly to undiffused fluorescent light, was greater than the real exposure to a larger extent in melanoma patients than in controls, and that this bias was stronger in the face to face interviews than on the subsequent postal questionnaires. Patients might tend to over-report any possible past exposure as compared to controls, but this would be expected to apply to outdoor exposure as well as to fluorescent light exposure and perhaps to occur similarly in direct interviews and in postal questionnaires. On direct questioning, very few of the participants interviewed admitted to any knowledge of an association between fluorescent light and melanoma.

Thus a bias originating in the subjects of the study seems unlikely. Bias due to the interviewers seems a more likely possibility. The interviewers were not aware of the case or control status of the interviewee subject at the beginning of the interview, but this information was divulged by about half the subjects before the end of the interview. To minimise bias, interviewers should not be involved in the design or interpretation of the study, but this ideal could not be met within our resources. Six different interviewers were used, and the detailed data concerning the differences between the results of interviews and postal questionnaires do suggest that the characteristics of the interviewer may be relevant. However, the numbers of subjects per interviewer are insufficient to support a firm conclusion.

The discrepancy between our results from interviews and from postal questionnaires is interesting when compared to other results on the same topic. The positive results initially recorded by Beral *et al.* (1982) were based on interviews, while of two studies showing in general no association between malignant melanoma and fluorescent light exposure, one was based on postal questionnaires in England (Sorahan & Grimley, 1985), and one on short telephone interviews given to subjects in Western Australia, who had been previously involved in a large study based on personal interviews but not mentioning fluorescent light (English *et al.*, 1985). In the United States, Dubin *et al.* (1985) report a study with findings similar to the current one, in that a significant positive association between melanoma and fluorescent light exposure was seen in the results of personal interviews, while a non-significant negative association was seen in data based on postal questionnaires sent subsequently to the same subjects. This difference arose because cases, but not controls, reported greater exposure to fluorescent light on interview than on postal questionnaires. Thus the question of whether results based on personal interviews may be producing bias in the direction of a spurious positive association, or whether results based on postal questionnaires or other short methods which are usually regarded as less reliable, are producing a spuriously weak result due to random errors, cannot be yet resolved.

Both in the study of Dubin *et al.* (1985) and in the current one, the differences in recorded sun exposure between interviews and questionnaires were much smaller than those with fluorescent light exposure, suggesting that the former is a less difficult item to recall accurately. The pattern of risk seen in the current study with outdoor occupational exposure on the postal questionnaires, with a high risk at moderate amount of exposure and a trend to lower risks at higher exposures is very similar to the pattern seen in the interview study of 595 case control pairs in Canada (Elwood et al., 1985b).

In regard to histories of exposure to other types of industrial lighting, the possibility of errors and bias in the responses is also high; patients with a serious disease might recall more readily occupational exposure on direct questioning, and we regard these results as preliminary and urge that they be further assessed. Their interpretation is also made difficult in that we were unable to directly confirm the precise nature of the lighting exposures, and as seen in the **Appendix**, the extent of exposure to some of these sources was small. However, in view of the importance of any such association, we recommend that further studies be performed.

We are grateful to the subjects and our interviewers, to Mrs Alison Langham for co-ordinating the questionnaire study, Mrs Joyce Gilbert for secretarial help, to Drs B.R. Allen and E. Saihan and Professor D.R. Turner for helpful discussions, and to the Nottingham Medical School Research Fund for financial help.

**Appendix** Specific occupational exposures reported by 83 melanoma cases and 83 matched controls

| *Sex* | *Age* | *Site* | *Exposure* | *Duration* | *Time* |
|---|---|---|---|---|---|
| *MELANOMA PATIENTS* | | | | | |
| Welding/foundry work | | | | | |
| M | 63 | Forehead | Red/white hot molten cast iron in foundry for 2–3 h day$^{-1}$ | 3 years | 1946–49 |
| M | 70 | Upper back | Arc welding, 1–3 ft distance, 1 h week$^{-1}$ | 18 years | 1945–83 |
| M | 51 | Upper back | Welding work | unknown, >year | unknown |
| M | 24 | Chest | Welding work | 6 years | 1975–81 |
| Film projection | | | | | |
| M | 63 | L. eyelid | Cinema projectionist; carbon arc bulb and mercury arc rectifier | 4 years | 1936–40 |
| M | 53 | L. forearm | Cinema projectionist; carbon arc lamp | 2 years | 1947–49 |
| Artillery photography | | | | | |
| M | 67 | Back | Gun tester, artillery. Bright bluish lamp used to photograph each round | 12 years | 1947–59 |
| M | 71 | Abdomen | Gun calibration, artillery. Flash spotting instrument | 6 years | 1940–46 |
| Spot lighting | | | | | |
| F | 42 | R. leg | Quartz hologen spot lamp for hair and cosmetic demonstrations | 10 years | 1974–83 |
| M | 39 | Back | High intensity spot lights in retail shop for 1 h day$^{-1}$ | 12 years | 1968–80 |
| M | 74 | Back | High intensity stroboscopic lamp to test objects in motion, 3 ft away | 42 years | 1930–72 |
| High intensity discharge lamps | | | | | |
| M | 40 | L. ear | Mercury vapour lamps in motor garage; also u.v. lamps in crack detection, gas welding | 17 years | 1966–83 |
| M | 24 | L. cheek | Sodium arc area lights; electric arc welding; also discotheque u.v. lights | 3 years | 1976–79 |
| M | 55 | Back | Sodium and mercury vapour lamps, | 2 years | 1948–50 |
| | | | and fixed u.v. lamp for security checks | 1 year | 1980–81 |
| Printing/dyeline copying | | | | | |
| F | 33 | R. arm | Printing/developing of plans | 1 year | 1968–69 |
| M | 67 | R. cheek | Carbon arc light and dyeline printing for plan copying, 1–2 h day$^{-1}$ | 25 years | 1950–75 |
| F | 43 | L. leg | Plan photography | 3 years | 1964–67 |
| Specific u.v. light sources | | | | | |
| F | 51 | R. leg | Insecticidal u.v. light in food shop; always right side | 11 years | 1972–83 |
| F | 18 | R. calf | u.v. light in catering dept. | 4 months | 1982 |
| F | 60 | Back | u.v. light in restaurant, 40 h day$^{-1}$, 8 ft away | 3 years | 1976–78 |
| M | 58 | Abdomen | u.v. light for metal crack detection, 1 h week$^{-1}$ | 30 years | 1939–70 |

| Sex | Age | Site | Exposure | Duration | Time |
|---|---|---|---|---|---|
| *CONTROL PATIENTS* | | | | | |
| Welding/foundry work | | | | | |
| M | 63 | | Acetylene and electric welding, 4 h day$^{-1}$ | 8 years | 1946–54 |
| F | 58 | | Gas welding | 2.5 years | 1942–45 |
| M | 37 | | Gas and arc welding | 9 years | 1973–84 |
| M | 34 | | Acetylene torch, 50 days total | <1 year | 1976–77 |
| M | 58 | | Arc welding | 13 years | 1960–73 |
| High density discharge lamps | | | | | |
| M | 24 | | Gas vacuum lamps | 2 years | 1981–83 |
| M | 70 | | Blue arc lamp in drawing office work | 5 years | 1928–34 |
| Specific u.v. light sources | | | | | |
| M | 55 | | u.v. lamp to match white cloth in bleaching, less than once/week | 40 years | 1943–83 |
| F | 48 | | u.v. germicidal lamp, food preparation, 5 ft away | 8 years | 1976–84 |
| Spot lighting | | | | | |
| F | 44 | | Intense white light to check flaws in glassware, 20 ft away, 40 h week$^{-1}$ | 2 years | 1963–65 |
| F | 55 | | Intense shop display white lights, 16 h week$^{-1}$ | 6 years | 1977–84 |

### References

BAKER, R.J. & NELDER, J.A. (1978). The GLIM System, Release 3. Generalized Linear Interactive Modelling. Numerical Algorithms Groups: Oxford.

BANKS, J.A. & INGRAM, J.A. (1983). Trent Regional Health Authority Patient Administration Computer Systems. *Medical Records and Health Care Information Journal,* **24,** 150.

BERAL, V. & EVANS, S. (1982). Malignant melanoma and exposure to fluorescence lighting at work. *Lancet,* **ii,** 1227.

BERAL, V., EVANS, S., SHAW, H. & MILTON, G. (1982). Malignant melanoma and exposure to fluorescent lighting at work. *Lancet,* **ii,** 290.

BERAL, V., EVANS, S., SHAW, H. & MILTON, G. (1983). Cutaneous factors related to the risk of malignant melanoma. *Br. J. Dermat.,* **109,** 165.

BRESLOW, N.E. & DAY, N.E. (1980). Statistical methods in cancer research. *IARC, Scientific Publications No. 32.* IARC: Lyon.

DUBIN, N., MOSESON, M. & PASTERNACK, B.S. (1985). Epidemiology of malignant melanoma: pigmentary traits, ultraviolet radiation and the identification of high risk populations. In *Epidemiology of Malignant Melanoma,* Gallagher, R.P. (ed). Springer-Verlag: Heidelberg (in press).

ELWOOD, J.M., GALLACHER, R.P., HILL, G.B., SPINELLI, J.J., PEARSON, J.C.G. & THRELFALL, W. (1984). Pigmentation and skin reaction to sun as risk factors for cutaneous melanoma: Western Canada Melanoma Study. *Br. Med. J.,* **288,** 99.

ELWOOD, J.M., GALLAGHER, R.P., DAVISON, J. & HILL, G.B. (1985*a*). Sunburn, suntan and the risk of cutaneous malignant melanoma. The Western Canada Melanoma Study. *Br. J. Cancer,* **51,** 543.

ELWOOD, J.M., GALLAGHER, R.P., HILL, G.B. & PEARSON, J.C.G. (1985*b*). Cutaneous melanoma in relation to intermittent and constant sun exposure. The Western Canada Melanoma Study. *Int. J. Cancer,* **35,** 427.

ENGLISH, D.R., ROUSE, I.L., XU, Z., WATT, J.D., HOLMAN, C.D.J., HENNAN, P.J. & ARMSTRONG, B.K. (1985). Cutaneous malignant melanoma and fluorescent lighting. *J. Natl. Cancer Inst.,* **74,** 1191.

GREEN, A., MACLENNAN, R. & SISKIND, V. (1985). Common acquired naevi and the risk of malignant melanoma. *Int. J. Cancer,* **35,** 297.

GREENE, M.H., CLARK, W.H., TUCKER, M.A., KRAEMER, K.H., ELDER, D.E. & FRASER, M.C. (1985). High risk of malignant melanoma in melanoma-prone families with dysplastic nevi. *Ann. Int. Med.,* **102,** 458.

HENDERSON, S.T. (1977). *Daylight and its Spectrum.* Hilger: London.

HOLMAN, C.D.J. & ARMSTRONG, B.K. (1984*a*). Pigmentary traits, ethnic origin benign nevi, and family history as risk factors for cutaneous malignant melanoma. *J. Natl. Cancer Inst.,* **72,** 257.

HOLMAN, C.D.J. & ARMSTRONG, B.K. (1984*b*). Cutaneous malignant melanoma and indicators of total accumulated exposure to the sun: an analysis separating histogenetic types. *J. Nat. Cancer Inst.,* **73,** 75.

KRAEMER, K.H., GREENE, M.H., TARONE, R., ELDER, D.E., CLARK, W.H. & GUERRY, D. (1983). Dysplastic naevi and cutaneous melanoma risk. *Lancet,* **ii,** 1076.

MAXWELL, K.J. & ELWOOD, J.M. (1983). UV radiation from fluorescent lights. *Lancet,* **ii,** 579.

MAXWELL, K.J. & ELWOOD, J.M. (1985). Could melanoma be caused by fluorescent light? A review of relevant physics. In *Epidemiology of Malignant Melanoma*. Springer-Verlag: Heidelberg (in press).

PASTERNACK, B.S., DUBIN, N. & MOSESON, M. (1983). Malignant melanoma and exposure to fluorescent lighting at work. *Lancet*, **i**, 704.

RIGEL, D.S., FRIEDMAN, R.J., LEVENSTEIN, M.J. & GREENWALD, D.I. (1983*a*). Malignant melanoma and exposure to fluorescent lighting at work. *Lancet*, **i**, 704.

RIGEL, D.S., FRIEDMAN, R.J., LEVENSTEIN, M.J. & GREENWALD, D.I. (1983*b*). Relationship of fluorescent lights to malignant melanoma: another view. *J. Dermatol. Surg. Oncol.*, **9**, 836.

SORAHAN, T. & GRIMLEY, R.P. (1985). The aetiological significance of sunlight and fluorescent lighting in malignant melanoma: a case-control study. *Br. J. Cancer* (in press).

SWERDLOW, A.J., ENGLISH, J., MACKIE, R.M., O'DOHERTY, C.J., HUNTER, J.A.A. & CLARK, J. (1984). Benign naevi associated with high risk of melanoma. *Lancet*, **ii**, 168.